# Professional Identity Formation in the model curriculum of human medicine in Oldenburg – a longitudinal approach

**DOI:** 10.3205/zma001832

**Published:** 2026-03-23

**Authors:** Kirsten Gehlhar, Michael Ankele, Imke Aits, Anne Dehlfing, Markus Ennen

**Affiliations:** 1 Carl von Ossietzky University Oldenburg, Faculty VI Medicine and Health Sciences, Dean’s Office of Studies, Oldenburg, Germany; 2 Carl von Ossietzky University Oldenburg, Faculty VI Medicine and Health Sciences, Department of General Practice, Oldenburg, Germany

**Keywords:** professional identity formation, professionalism, reflection, feedback, tutor, CanMEDS

## Abstract

**Background:**

Professional identity formation (PIF) in medical education is a complex and transformative process that aims to enable students to meet the expectations of medical integrity and societal responsibility. It involves the acquisition of necessary competencies and the development of a professional identity. Teaching this aspect remains challenging – even in competency-based curricula. Methods such as portfolios, reflective writing, and coaching are used to promote long-term identity development and awareness of the physician’s role.

**Project description:**

In 2012, a longitudinal track titled “Professional Identity Formation” (PIF) was established at the University of Oldenburg, modelled on a program at its partner institution in Groningen. Early student evaluations indicated that the relevance of this track was often questioned – particularly in the early years of study. As a result, a substantial reform was implemented in the winter semester 2021/22. Current evaluation results show increased acceptance: 74% of surveyed students (n=203) now consider PIF to be well integrated into the overall curriculum. Nonetheless, the success of the program still strongly depends on the engagement and quality of the assigned tutors.

**Conclusion:**

Competency-based support for professional identity formation is a challenging endeavour that depends not only on the structural and didactic design of the curriculum but also on students’ willingness to engage with reflective methods and topics. The implementation of the program by tutors – despite extensive training – remains a critical but hard-to-control factor. However, the effectiveness of the PIF track depends heavily on these instructors.

## Zusammenfassung

**Hintergrund:**

Professionelle Entwicklung im Medizinstudium ist ein komplexer, transformativer Prozess, der Studierende dazu befähigen soll, die Anforderungen an ärztliche Integrität und gesellschaftliche Verantwortung zu erfüllen. Sie beinhaltet den Erwerb von notwendigen Kompetenzen und die Entwicklung einer beruflichen Identität. Dies zu unterrichten ist jedoch auch im Rahmen kompetenzbasierter Curricula nicht einfach. Methoden wie Portfolios, reflektierendes Schreiben und Coaching sollen die langfristige Professionalitätsbildung und Sensibilisierung für die ärztliche Rolle fördern.

**Projektbeschreibung:**

2012 wurde der longitudinalen Pfad „Professionelle Entwicklung“ (PE), nach dem Vorbild der Partner-Universität in Groningen etabliert. Da die studentischen Evaluationsergebnisse zeigten, dass die Sinnhaftigkeit des Pfades gerade in den ersten Studienjahren häufig infrage gestellt wurde, erfolgte zum WiSe 2021/22 eine weitgehende Reform. Aktuelle Evaluationsergebnisse zeigen, dass die Akzeptanz nach der Reform gestiegen ist. 74% der befragten Studierenden (n=203) sehen eine gute Einordnung von PE im Gesamtcurriculum. Trotz dessen gibt es nach wie vor eine starke Abhängigkeit des Erfolgs von den betreuenden Tutor:innen.

**Schlussfolgerung:**

Die kompetenzbasierte Unterstützung bei einer professionellen Identitätsentwicklung ist ein schwieriger Prozess, der auch von der Bereitschaft der Studierenden abhängt, sich auf Methoden wie Reflexion und Feedback und die behandelten Themen einzulassen. Die Umsetzung des Konzepts durch die Tutor:innen ist – auch mit vielen Schulungen - ein nur schwer zu kontrollierender Faktor. Der Erfolg hängt jedoch maßgeblich von diesen Dozierenden ab.

## 1. Introduction

Professional identity formation (PIF) in medical students describes a long-term, dynamic process in which future physicians align their personal identity with the professional role within the medical context [1], [2], [3]. This process involves the gradual internalization of professional values, norms, and behaviour, the acquisition of profession-specific attitudes, and the development of a professional self-concept. PIF is shaped significantly by social interactions, clinical experiences, role models, and structured reflection processes. The resulting professional identity is not solely based on medical expertise, but also integrates ethical, communicative, and psychosocial dimensions of medical practice [3], [4].

In contrast, institutional professionalism refers to the expectations of professional conduct shaped by occupational, legal, and organizational frameworks. It is based on an implicit social “contract” between the medical profession and society that defines competencies, responsibilities, ethical standards, and scopes of action [5]. In a contractualistic sense, institutional professionalism is guided by the normative principle that physicians should act in the interest of the public good, scientific reasoning, and ethical integrity – in return for professional autonomy and public trust [6], [7].

While institutional dimensions of professionalism are primarily addressed in courses such as “history, theory, and ethics of medicine” or medical law, formats focused on professional identity formation aim to accompany students throughout their medical education. These formats are designed to help students acquire not only the skills needed to navigate an academically rigorous and clinically complex curriculum, but also the competencies necessary to responsibly fulfil diverse medical roles and develop a reflective professional identity [8]. That professionalism should be taught in medical school was recommended as early as 1999 by the Association of American Medical Colleges (AAMC), reaffirmed in 2010 by the LCME, and formally integrated into the CanMEDS competency framework developed in 2000 [9]. Based on the CanMEDS roles, the University of Groningen had already defined seven roles for medical students in its 2010 curriculum (G2010), explicitly including reflection as a central element. Mere role modelling without guided reflection – where students learn solely by observing experienced professionals – was long the norm but is now considered insufficient in competency-based curricula [10], even though positive role models and visible professionalism continue to play a key role for students [11]. The PIF track developed in Groningen was adopted in principle when the medical program in Oldenburg was founded.

Various approaches have been proposed to support the professional identity formation of medical students. These range from systemic constellation methods (to raise awareness of the socialization process [12]) to more common approaches such as dedicated modules [13] or longitudinal tracks [1], [14], [15], [16], in which methods such as e-portfolios, reflective writing, experiential learning, and coaching are employed.

Particularly effective are:

1. Situated learning [17], [18], in which role models play a central role [19], [20], [21]
2. Reflection on one’s own experiences, e.g., through portfolios or short written or oral reports [22], [23], [24], which are among the most frequently described methods in the literature [25].

Both approaches benefit from small group formats [26], [27] and from physician tutors who support students in their reflective and learning processes [28].

These methods are also used in the longitudinal “Professional Identity Formation” (PIF) track, which has been a core element of the Oldenburg model curriculum since the medical program was launched in 2012. It accompanies students from the first to the final semester and is designed to promote awareness of one's actions, decision-making, and role understanding. The concept was adapted from the partner university in Groningen and integrated into the curriculum in Oldenburg [29].

A key focus of the program is the reflection on the so-called physician roles or key competencies described in CanMEDS [9], which represent the core requirements for medical professionals in daily practice.

The objective of this project report is to present the longitudinal PIF track, describe the curriculum revision carried out in 2021, and compare evaluation results from before and after the reform.

## 2. Project description

### 2.1. Setting

The model curriculum in human medicine at the European Medical School Oldenburg-Groningen was launched in the winter semester of 2012/13 and currently offers 120 study places per year. The program pursues innovative approaches in the training of medical students and already implements many of the core goals of the Master Plan 2020 and the National Competency-Based Learning Objectives Catalogue for Medicine 2.0 (German: NKLM), such as competency-based education, patient-centredness, doctor-patient communication, vertical and horizontal integration, the promotion of scientific practice, strengthening of general practice, and inter-professional teaching [30]. At the same time, the program aims to promote European cooperation in medical education through its partnership with Groningen. A particular feature of the curriculum is the integration of several longitudinal tracks that run parallel to the thematic modules throughout the entire program. One of these is the mandatory longitudinal track “Professional Identity Formation” (PIF), which extends across all years of study.

The sessions in the “Professional Identity Formation” (PIF) track take place in small groups of up to ten students. These groups meet eight times per year for sessions of three hours each, under the guidance of a physician tutor. At the end of each academic year, the groups are deliberately rearranged to allow for new perspectives and group dynamics. For each year of study, there is a handbook for students and one for tutors, in which each PIF session is described in terms of background, objectives, and content. In addition, these handbooks contain a toolbox with didactic guidance and supplementary materials (e.g., feedback rules, assessment criteria, and ideas for structuring sessions, such as think-pair-share [31], placemat [32], flashlight round [33], Kaluza’s stress traffic light [34], six-step problem-solving method [34]).

All assignments, written reflections, and formative feedback from clinical supervisors are collected in individual student portfolios. At the end of the academic year, the tutors review the portfolios with each student individually and assess them on a pass/fail basis. Tutors are recruited from the medical teaching staff of the faculty, and regular calls are issued inviting faculty members to volunteer as PIF tutors. Tutors receive training before conducting their first session, and regular networking meetings are held. Tutor training includes an introduction to the PIF curriculum (overview, structure and format, content, didactic guidance, and assessment criteria). The role of the PIF tutor is also explicitly addressed and discussed.

## 3. Structure of the Professional Identity Formation track

### 3.1. Initial curriculum 2012-2020

In the first three academic years, students were expected to develop an awareness of their own actions, decision-making processes, and understanding of their professional role. Through reflection on these elements, they were to learn how to justify and further develop their professional behaviour. In each session, predefined tasks from the handbooks addressing aspects of professional identity were completed and discussed. For example, the one-week general practice internships were prepared for and debriefed in PIF sessions, with a particular focus on communication. Students also reflected collectively on how the supervising physicians fulfilled the CanMEDS roles and how they themselves experienced these roles. In addition, each year, students completed a “career profile” regarding their interests, in order to track changes over time. In the second part of the sessions, students discussed predefined thematic topics together.

In the fourth year of study, students were expected to further develop their awareness of their actions, decisions, and role behaviour. This included analyzing personal strengths and weaknesses and developing strategies to address problems with colleagues constructively. Topics drawn from clinical practice – such as stigmatization, patient safety, or cultural diversity – were addressed. Moreover, a bridge was established between the students’ early clinical experiences in internships and the insights they could derive from these for their ongoing development. In each session, half of the time was allocated to topics brought in by students – issues they encountered in the clinical environment that they wished (or were expected) to reflect on in order to develop a professional approach. A topic repository provided further ideas if students had no suggestions of their own.

In the fifth year of study, the longitudinal tracks “communication”, “research”, and “Professional Identity Formation” were merged within the Oldenburg curriculum. The program consisted of portfolio assignments, a 1:1 mentoring program, and a broad, semi-elective workshop curriculum covering areas such as communication, clinical skills, physician roles, and research. Students were free to choose offerings that suited their needs. Individual mentoring was part of the PIF program in year 5.

In the sixth year of study, the PIF track was limited to assignments included in the final-year logbooks (PJ). Supervising physicians took on the role of PIF tutors. At the beginning, middle, and end of each rotation (Tertial), students were expected to reflect on and assess their abilities within the CanMEDS roles. At the end of the final year, they submitted a brief reflection on their development in these roles and formulated personal goals for the future.

### 3.2. Evaluation results of the initial track

The results from student evaluations conducted between 2012 and 2014 are presented in attachment 1 . The questionnaires were revised several times, which led to different sets of questions and scaling formats. As a result, the findings are not directly comparable. The surveys were conducted using paper questionnaires. Between 2016 and 2020, no specific evaluations of the format were carried out, and no data are available for that period.

In the tables, each survey includes information on the response rate (RR), the number of completed questionnaires (n), and the number of students enrolled in the format (N). For each item, the mean (M), number of responses (n), and standard deviation (SD) are provided. Ordinal Likert scales were treated as interval scales. For some evaluations in 2013/14, only summary reports were available, no raw data, making aggregation of results impossible. Free-text responses were grouped by content using inductive coding.

Satisfaction with the PIF track in the early years of the program varied greatly, with significant fluctuations in the ratings of both the format (German school grades 2.3 to 3.6; 1 being the best and 5 being the worst result) and the tutors (1.8 to 3.8), depending on module and cohort (see attachment 1 ). Due to the changing questionnaires, a combined analysis of individual items across years is not feasible. However, insights can be drawn from the 371 free-text comments from 2013 to 2015. Of these, 303 were critical, while 53 were positive.

Table 1 (Tab. 1) below provides an overview of the summarized critical free-text comments (all categories with n≥5), including one original quote as an example (translated from German).

Positive remarks highlighted the group atmosphere, the tutors, the reflection on practical experiences, and the opportunity for self-reflection (see table 2 (Tab. 2)).

### 3.3. Curriculum revision for 2020/21

In response to feedback from students and tutors, a comprehensive revision of the PIF track was carried out in spring 2021. In addition to refining the conceptual focus, greater alignment with the NKLM (German abbreviation for “National Competency-Based Learning Objectives Catalogue for Medicine”) was sought. The initial track had not been based on the NKLM, as the Groningen curriculum, which served as a blueprint, was structured around the CanMEDS roles rather than the NKLM. Specifically, the revised version incorporated the following learning goals:

- Understanding and practicing self-reflection and self-criticism
- Recognizing and respecting personal limits
- Developing intra-, inter-, and multi-professional collaboration skills
- Considering ethical aspects, values, and norms in medical practice
- Considering social, cultural, ethnic, religious, age-, gender-, and disability-related aspects of patients
- Maintaining and improving one’s own professional conduct as a lifelong learner
- Reflections on work-life balance and career planning

For the winter semester 2021/22, a task force was established to revise the concept. It included members from the Dean’s Office of Studies, the departments of general practice and ethics in medicine, as well as student representatives.

The revised concept retained the small-group format (ten students with one physician tutor) as its core teaching method, and the PIF track continued to be embedded longitudinally across the curriculum.

Each academic year was assigned a thematic focus:

- Year 1: Profession and professionalism
- Year 2: Societal and structural conditions of medical practice
- Year 3: Preparation and reflection on clinical experiences
- Year 4: Reflection on clinical experiences
- Year 5: Supporting students in the transition from university to clinical practice

In addition, all academic years include sessions on *health promotion and self-care.* This element was considered essential to the track, as medical students, both nationally and internationally, show high prevalence rates of depression and increased incidences of burnout [35], [36]. Personal self-care is thus a core component of professional medical behaviour and is addressed and supported throughout the PIF curriculum. The ability of future physicians to manage stress – i.e., their resilience – is largely shaped during their studies and in the years preceding them [37], [38].

- *Year 1:* In the first year of the PIF track, students are introduced to the concept of professional identity formation. Two one-week general physician internships embedded in the curriculum are prepared and debriefed in group sessions, focusing on reflections about the professional conduct of the supervising general practitioners. Initial elements of health promotion and self-care are introduced during a plenary session held for the entire cohort.
- *Year 2:* The second year focuses on inter-professionalism. Students learn about the relevance of cooperation with other health professions in healthcare. Additional topics include health promotion, self-care, and legal aspects of the medical profession.
- *Year 3: *In the third year, the focus shifts to sensitivity toward diversity. Different forms (e.g., racism, sexism, ableism) and levels (direct, indirect, structural) of discrimination in medicine – and one’s own involvement in such structures – are addressed. A further topic is the critical examination of economic factors in medical practice and the commercialization of healthcare.
- *Year 4: *The clinical component of the curriculum increases significantly in the fourth year. In the PIF groups – as in the previous concept – challenges encountered in clinical settings are discussed using structured peer case consultation (“kollegiale Fallberatung”[39]). Additionally, students can draw on topics from a shared repository (e.g., child protection, self-reflection, professional profiling) to relate them to their own clinical experiences. Students are encouraged and enabled to engage with these issues in a safe setting, develop their own positions, and explore possible courses of action through discussion.
- *Year 5:* In the fifth year, the focus is on the transition from undergraduate studies to professional practice. Due to the growing cohort size, the previous 1:1 mentoring program was replaced with small-group formats. Four group sessions address reflection on the CanMEDS roles, resilience, self-care, and the handling of professional responsibility and potential ethical dilemmas.

In addition, consultations and workshops are offered for students to discuss individual questions – e.g., about the research project or the final clinical year (PJ) – in a 1:1 format with their tutors.

The Year 5 workshop program continues to form a second key component of the PIF track.

In all years of study, the reflection on assumptions, observations, and experiences in relation to physician roles serves as a guiding principle for the development of professional behaviour. The handbooks continue to define the portfolio tasks, which are assessed by the tutors at the end of each academic year.

### 3.4. Evaluation of the revised PIF track

The available evaluation results for the first cohorts following the reform suggest that the sharpening of the track’s profile was successful – students questioned the meaningfulness of the format significantly less often than earlier cohorts. Student criticism now focused more on content-related aspects, organizational issues, or on individual tutors.

Table 3 (Tab. 3) presents a summary of selected critical free-text comments (multiple mentions) from the 2024 evaluation (n=198).

Table 4 (Tab. 4) presents the positive free-text comments.

In the current questionnaires, students frequently named student-related topics as their preferences. The topic of self-care (mentioned 7 times) was especially popular in years 2 and 3; in the final year, professional perspectives (4 mentions) were most relevant. Requests for meta-medical topics (6 mentions) were broad and varied.

In addition, the year 5 workshop program received notably better evaluations. Many students appreciated the opportunity to discuss difficult situations, and both the topics and instructors of the workshops were rated very positively (see attachment 2 and attachment 3 ). The mentoring element in year 5 was also well received. A majority of students perceived it as personally beneficial (see attachment 2 ).

Evaluation results from 2021 to 2024 after the reform are presented in attachment 2 , attachment 3 and attachment 4 . These surveys were conducted online. The current version of the evaluation questionnaire for the PIF format is included in attachment 5 .

## 4. Discussion

Since 2015, the National Competency-Based Learning Objectives Catalogue for Medicine (German abbreviation “NKLM”) [https://nklm.de/zend/menu] has recommended that professional behaviour and professional self-reflection be systematically integrated into medical curricula as core competencies of medical practice. Increasingly, faculties have been implementing this recommendation [14], [15], [16], [39], [40]. While the targeted support and facilitation of students’ professional identity formation is meaningful and desirable, its implementation has proven to be complex and multifaceted.

Our results show a broad range of student perspectives on the PIF track. While some students find the peer exchange enriching, others express clear reservations. Critical feedback often relates to the lack of relevance to exams, the perceived workload, and the difficulty of opening up in group settings. A recurring concern is the limited opportunity for students to bring in their own topics. In contrast, this opportunity is positively highlighted during the clinical phase of the curriculum starting in year 4. Tutors play a central role in the effectiveness of the program, but are also perceived in very different ways.

This wide spectrum of responses – from enthusiastic acceptance to clear rejection – has also been observed in comparable studies [16]. Differences can, among other factors, depend on students’ backgrounds (e.g., previous vocational training [16], although no such effect was observed in our data) or on their learning styles. Students who prefer teacher-centred learning approaches often find it more difficult to engage with reflective formats [19], [41]. Demonstrating the personal benefit of PIF for lifelong learning and a reflective, self-critical approach to the medical profession [42] is not always easy, in our view. This may be because students already experience the program as time-consuming and stressful. Under these circumstances, motivating all students to engage with topics that are not directly relevant for exams – and whose benefits may not be immediately obvious – remains a challenge for both curriculum developers and tutors.

Implementation appears to be more successful in areas where students perceive direct benefit and when topics are aligned with their own interests. For example, the increase in approval ratings in the clinical years (see attachment 3 , Item 4.1) may be interpreted to indicate that students experience the value of reflection more tangibly as their studies progress. In years 4 and 5, clinical internships increasingly provide real-life reference points for PIF content (see attachment 3 , Items 4.2, 4.3 and 9.1), which may also lead to better evaluations. The opportunity to address personal topics may further contribute to this approval. The topic of resilience is clearly important to students and frequently appears in free-text responses. Literature and our own faculty’s experience show that medical students increasingly experience stress and a decline in mental health during their studies – and that these negative developments begin early [43], [44]. Promoting resilience is therefore essential for improving student well-being, supporting the health of future physicians [45], [46], and ensuring high-quality patient care [47].

Another frequently mentioned criticism in free-text responses is the lack of opportunity to contribute personal topics. This possibility is formally incorporated into the PIF track starting in year 4. This may further explain why approval of PIF increases from that point onward. However, from the perspective of curriculum developers, students are also expected to engage with predefined topics – even if this conflicts with their desire for autonomy. In our view, personal topics alone are not always sufficient to achieve the goals of PIF.

To provide more space for discussing students’ individual concerns and to relieve the PIF track of this task, the faculty established a peer mentoring program in 2023 [https://uol.de/fk6/studium-lehre/mentoring]. This program offers a dedicated space for personal questions and issues (e.g., learning strategies), thus supporting students outside the formal PIF structure.

One important step taken in the revision of the program at the University of Oldenburg was the active involvement of students in the redesign process.

Tutors are responsible for initiating group discussions, motivating students to participate, and managing challenging group dynamics. Literature emphasizes the importance of a safe and supportive learning environment for reflection [48], [49], [50], [51], which places high demands on tutors. Poorly executed mentoring roles can negatively affect students’ learning experiences [52], [53]. The influence of the tutor is consistently described as central – both in our results and in the literature – yet it is difficult to control and strongly dependent on the individual tutor’s attitude and prior experience [54], [55], [56], [57], [58]. Conversely, tutors can also benefit from their role in the PIF track. They report personal and professional development and increased reflection on their own values and teaching practices [59], [60]. Although we have made efforts to prepare tutors through regular (non-mandatory) training sessions, their influence on the success of the sessions remains considerable, difficult to control, and one of the key factors that students consistently rate as either very good or very poor. This raises the question of whether mandatory training could improve tutor quality. On the other hand, making training obligatory could reduce the number of willing tutors.

### 4.1. Limitations

The findings and experiences presented here are based on a specific medical faculty and may not be readily transferable to other universities or countries. Furthermore, the curriculum reform was based on student feedback, which is inherently subjective and may not capture all relevant aspects. Since the evaluation instruments were repeatedly modified during the early years of the PIF track, the resulting data cannot consistently be linked or compared over time. As such, it is currently not possible to assess the long-term impact of the changes on students’ professional identity formation using these tools. Such assessments will only be possible in the coming years. Response rates for the evaluations in 2021 and 2023 were also very low, which calls the representativeness of the results into question.

Moreover, shifts in student perception regarding the PIF track may not be attributable solely to the curricular reform but also to external influences (e.g., societal trends, other teaching formats).

## 5. Conclusions

The professional identity formation of medical students is a complex, individual process that requires time, space, and appropriate structures. Our findings demonstrate that programs designed to support this development receive widely varying responses and depend heavily on the quality of tutor facilitation. Increasing opportunities for students to contribute their own topics, and linking the sessions more closely to clinical experiences, may help increase both the perceived relevance and the acceptance of the program. In the long term, it would be desirable to investigate retrospectively whether the PIF track has a sustained effect on graduates’ professional practice. Despite the challenges, the positive feedback shows that a structured program to support professional identity formation is worthwhile and should be further developed.

## Acknowledgements

We would like to thank Prof. J.B.M. Kuks from Groningen for his support in implementing the Professional Identity Formation track and for his assistance in developing the first version of the PIF handbooks for Oldenburg.

We also thank Hanke Dekker and Menno de Bree (also from Groningen) for conducting the initial tutor trainings.

Our thanks also go to the task force responsible for the revision of the track. In addition to the co-authors, this included the Department of General Practice (Prof. Michael Freitag, Sabine Kurpgoweit, Prof. Bettina Engel) and the Department of Ethics in Medicine (Prof. Mark Schweda, Niklas Ellerich-Groppe, and Simon Gerhards).

## Authors’ ORCIDs

- Kirsten Gehlhar: [0009-0003-1456-9046]
- Michael Ankele: [0009-0007-5604-4028]
- Imke Aits: [0009-0007-6118-1705]

## Competing interests

The authors declare that they have no competing interests. 

## Supplementary Material

Evaluation results 2012/13 – 2014/15

Survey of study years 1 and 1-4 (2021 and 2023)

Survey on PIF year 5 in 2021 and 2023

Evaluation of PIF 1-5 (2024)

Current questionnaire

## Figures and Tables

**Table 1:**
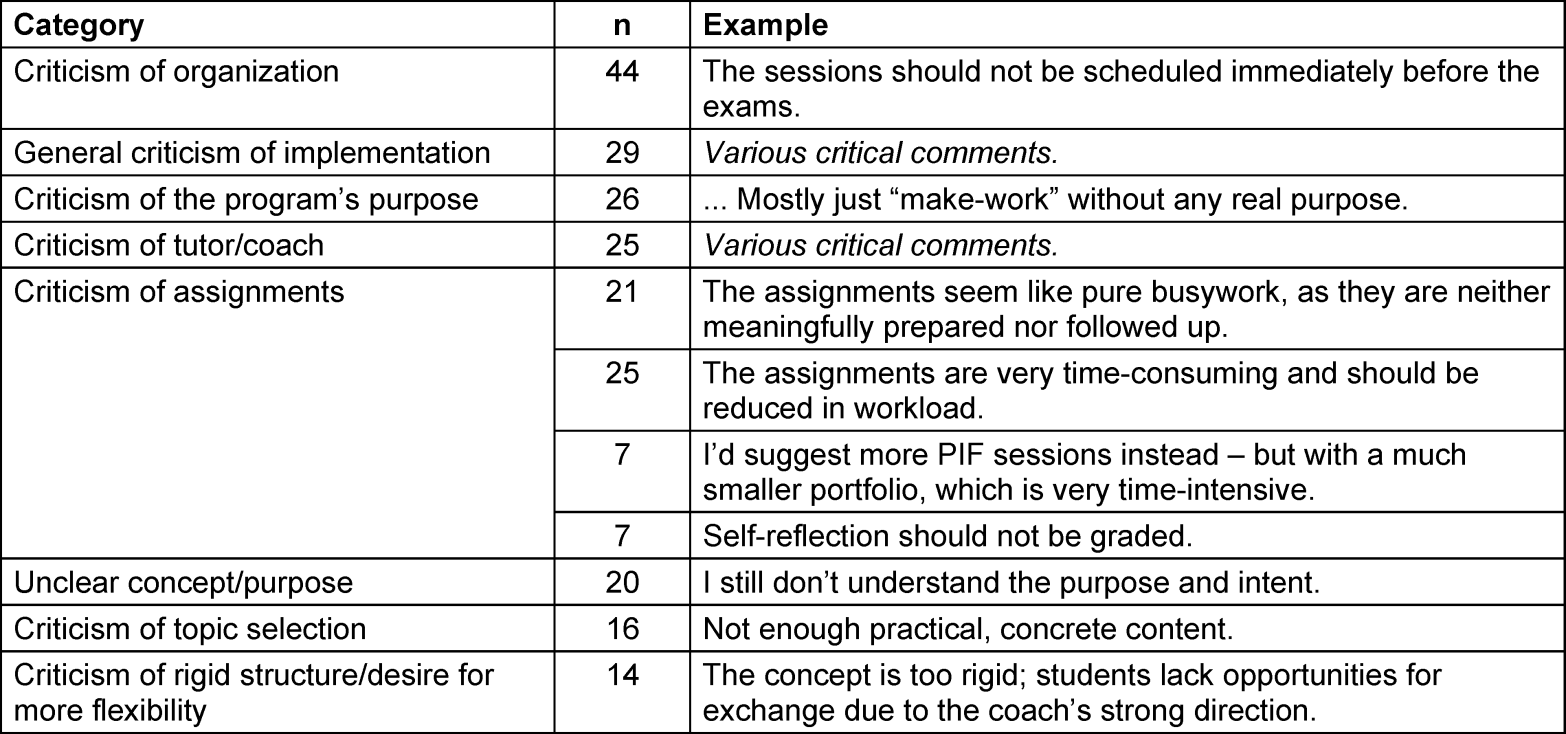
Summary of critical free-text comments from 2012-2014 (inductive coding), each with one translated original quote as an example

**Table 2:**
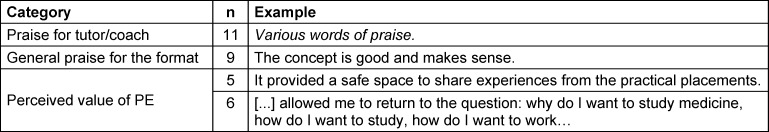
Summary of positive free-text comments from 2012-2014 (inductive coding), each with one translated original

**Table 3:**
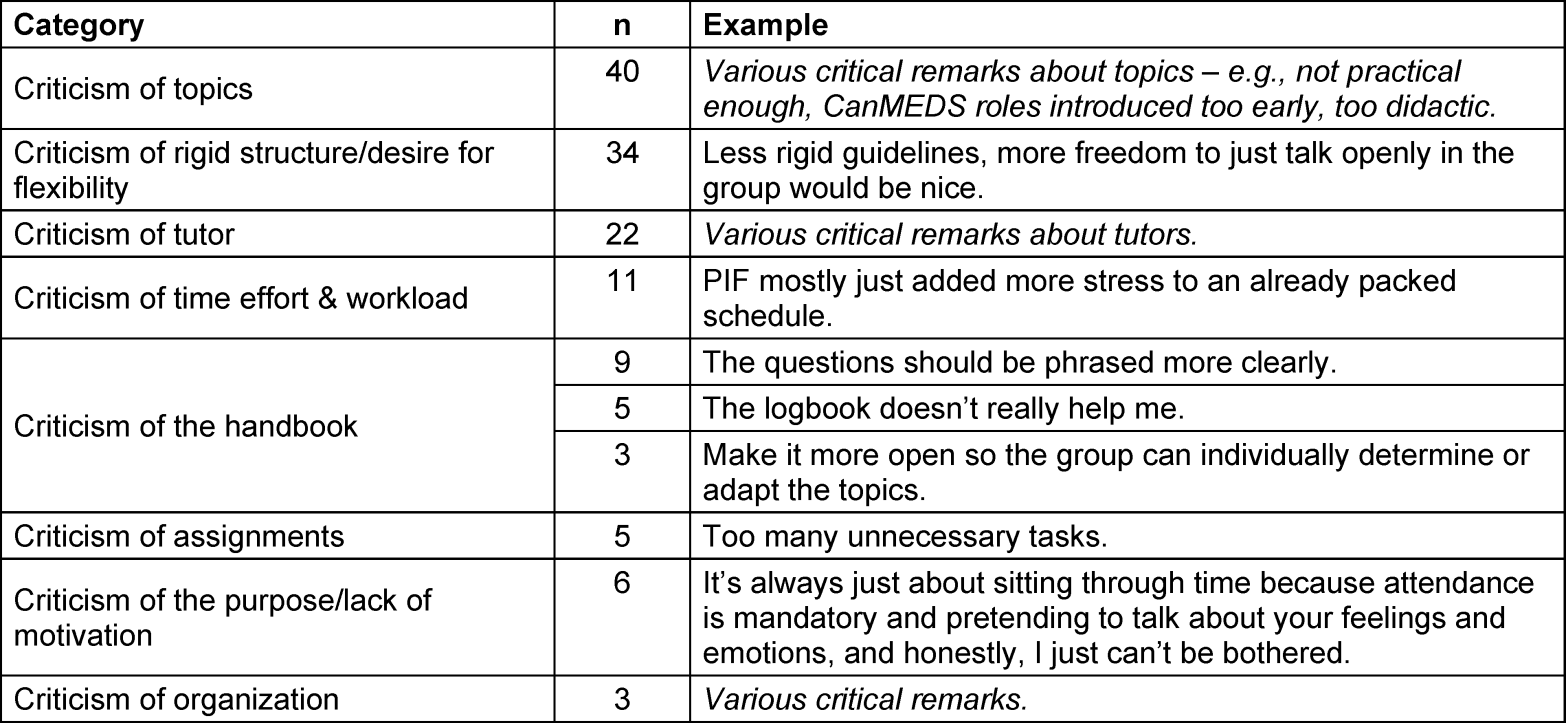
Summary of critical free-text comments from 2024 (inductive coding), each with one translated original quote as an example

**Table 4:**
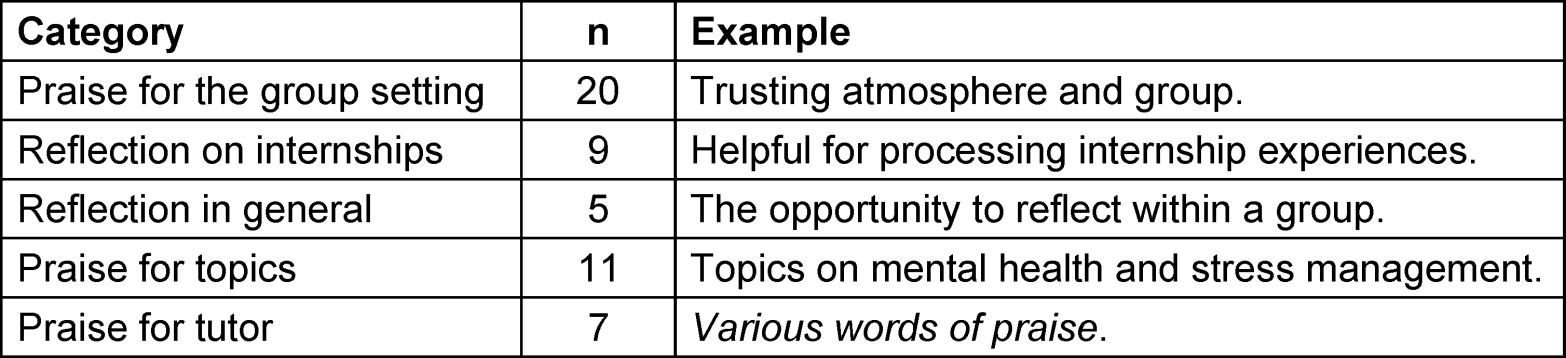
Summary of positive free-text comments from 2024 (inductive coding), each with one translated original quote as an example

## References

[1] Wald HS, Anthony D, Hutchinson TA, Liben S, Smilovitch M, Donato AA (2015). Professional identity formation in medical education for humanistic, resilient physicians: pedagogic strategies for bridging theory to practice. Acad Med.

[2] Holden MD, Buck E, Luk J, Ambriz F, Boisaubin EV, Clark MA, Mihalic AP, Sadler JZ, Sapire KJ, Spike JP, Vince A, Dalrymple JL (2015). Professional identity formation: creating a longitudinal framework through TIME (Transformation in Medical Education). Acad Med.

[3] Jarvis-Selinger S, Pratt DD, Regehr G (2012). Competency is not enough: integrating identity formation into the medical education discourse. Acad Med.

[4] Cruess RL, Cruess SR, Boudreau JD, Snell L, Steinert Y (2015). A schematic representation of the professional identity formation and socialization of medical students and residents: a guide for medical educators. Acad Med.

[5] Siepmann M, Groneberg DA (2012). Der Arztberuf als Profession — die strukturtheoretische Perspektive. Zbl Arbeitsmed.

[6] Cruess RL, Cruess SR (2008). Expectations and obligations: professionalism and medicine's social contract with society. Perspect Biol Med.

[7] Dekker H (2014). Teaching and learning professionalism in medical education. Thesis fully internal (DIV).

[8] Chen S, Traba C, Lamba S, Soto-Greene M, Gotian R, Yang Y, Safdieh J (1999). Professional and Career Development of Medical Students.

[9] Frank JR, Jabbour M, Tugwell P (2000). CanMEDS 2000: extract from the CanMEDS 2000 societal needs working group report. Med Teach.

[10] Stern DT (2003). Can professionalism be taught?. Virtual Mentor.

[11] Byszewski A, Hendelman W, McGuinty C, Moineau G (2012). Wanted: role models--medical students' perceptions of professionalism. BMC Med Educ.

[12] Scholtens S, Boer H, Smit M, Stam JJ, Barnhoorn PC, Schmitt A, Fleer J (2020). Introduction of a new teaching method to support Professional Identify Formation in medical students. Research Square.

[13] Shrivastava SR, Shrivastava PS (2023). Creating a professional development plan for medical teachers: Need of the hour. J Clin Sci Res.

[14] Zupanic M, Schulte H, Ehlers JP, Haller J, Kiessling C, Krämer M, Zumbach J, Deibl I (2020). Konzept zur interprofessionellen Persönlichkeitsentwicklung im Modellstudiengang 2018+: Auf dem Weg zu meiner beruflichen Identität!.

[15] Schmidt K, Siller K, Rißmann J, Andlauer M, Feustel J, Klein F, Petruschke I, Schulz S (2024). Professional development of medical students - piloting a longitudinal curriculum at Jena University Hospital (LongProf). GMS J Med Educ.

[16] Schrempf S, Herrigel L, Pohlmann J, Griewatz J, Lammerding-Köppel M (2022). Everybody is able to reflect, or aren't they? Evaluating the development of medical professionalism via a longitudinal portfolio mentoring program from a student perspective. GMS J Med Educ.

[17] Cruess RL, Cruess SR (2006). Teaching professionalism: general principles. Med Teach.

[18] Maudsley G, Strivens J (2000). Promoting professional knowledge, experiential learning and critical thinking for medical students. Med Educ.

[19] Cruess SR, Cruess RL, Steinert Y (2019). Supporting the development of a professional identity: General principles. Med Teach.

[20] O'Sullivan H, van Mook W, Fewtrell R, Wass V (2012). Integrating professionalism into the curriculum: AMEE Guide No. 61. Med Teach.

[21] Passi V, Johnson S, Peile E, Wright S, Hafferty F, Johnson N (2013). Doctor role modelling in medical education: BEME Guide No. 27. Med Teach.

[22] Driessen E, van Tartwijk J, van der Vleuten C, Wass V (2007). Portfolios in medical education: why do they meet with mixed success? A systematic review. Med Educ.

[23] Challis M (1999). AMEE Medical Education Guide No.11 (revised): Portfolio-based learning and assessment in medical education. Med Teach.

[24] Branch WT (2005). Use of critical incident reports in medical education. A perspective. J Gen Intern Med.

[25] Mount GR, Kahlke R, Melton J, Varpio L (2022). A Critical Review of Professional Identity Formation Interventions in Medical Education. Acad Med.

[26] Schaub-de Jong MA, Cohen-Schotanus J, Dekker H, Verkerk M (2009). The role of peer meetings for professional development in health science education: a qualitative analysis of reflective essays. Adv Health Sci Educ Theory Pract.

[27] Henderson E, Berlin A, Freeman G, Fuller J (2002). Twelve tips for promoting significant event analysis to enhance reflection in undergraduate medical students. Med Teach.

[28] Sandars J (2009). The use of reflection in medical education: AMEE Guide No. 44. Med Teach.

[29] Kuks J (2019). The bachelor-master structure (two-cycle curriculum) according to the Bologna agreement: a Dutch experience. TS Medisch Onderwijs.

[30] Gehlhar K (2019). The model medical degree programme "human medicine" in Oldenburg - the European Medical School Oldenburg-Groningen. GMS J Med Educ.

[31] Bönsch M (2002). Basiswissen Pädagogik - Unterrichtskonzepte und -techniken.

[32] Mattes W (2011). Methoden für den Unterricht.

[33] Paradies L, Linser HJ (2019). Differenzieren im Unterricht.

[34] Kaluza G (2018). Stressbewältigung: Trainingsmanual zur psychologischen Gesundheitsförderung.

[35] Rotenstein LS, Ramos MA, Torre M, Segal JB, Peluso MJ, Guille C, Sen S, Mata DA (2016). Prevalence of Depression, Depressive Symptoms, and Suicidal Ideation Among Medical Students: A Systematic Review and Meta-Analysis. JAMA.

[36] Erschens R, Keifenheim KE, Herrmann-Werner A, Loda T, Schwille-Kiuntke J, Bugaj TJ, Nikendei C, Huhn D, Zipfel S, Junne F (2019). Professional burnout among medical students: Systematic literature review and meta-analysis. Med Teach.

[37] Dunn LB, Iglewicz A, Moutier C (2008). A conceptual model of medical student well-being: promoting resilience and preventing burnout. Acad Psychiatry.

[38] Slavin SJ, Chibnall JT (2016). Finding the Why, Changing the How: Improving the Mental Health of Medical Students, Residents, and Physicians. Acad Med.

[39] Frei E, Stamm M, Buddeberg-Fischer B (2010). Mentoring programs for medical students--a review of the PubMed literature 2000-2008. BMC Med Educ.

[40] Meinel FG, Dimitriadis K, von der Borch P, Störmann S, Niedermaier S, Fischer MR (2011). More mentoring needed? A cross-sectional study of mentoring programs for medical students in Germany. BMC Med Educ.

[41] Driessen E (2017). Do portfolios have a future?. Adv Health Sci Educ Theory Pract.

[42] Mann K, Gordon J, MacLeod A (2009). Reflection and reflective practice in health professions education: a systematic review. Adv Health Sci Educ Theory Pract.

[43] Kötter T (2019). Ansatzpunkte für Resilienzförderung im Medizinstudium – Was hält angehende Ärztinnen und Ärzte gesund?. Akt Urologie.

[44] Scholz M, Neumann C, Steinmann C, Hammer CM, Schröder A, Eßel N, Paulsen F, Burger PH (2015). Entwicklung und Zusammenhang von Arbeitsverhalten, Burnout-Beschwerden und Lebensqualität bei Studierenden der Humanmedizin vom Studienstart bis zum ersten Staatsexamen. Psychother Psychosom Med Psychol.

[45] Rönnau-Böse M, Fröhlich-Gildhoff K, Rönnau-Böse M (2021). Resilienzförderung von Studierenden.

[46] Epstein RM, Krasner MS (2013). Physician resilience: what it means, why it matters, and how to promote it. Acad Med.

[47] Peters D, Horn C, Gishen F (2018). Ensuring our future doctors are resilient. BMJ.

[48] Moos RH (1973). Conceptualizations of human environments. Am Psychol.

[49] Schönrock-Adema J, Bouwkamp-Timmer T, van Hell EA, Cohen-Schotanus J (2021). Key elements in assessing the educational environment: where is the theory?. Adv Health Sci Educ Theory Pract.

[50] Schönrock-Adema J, Visscher M, Raat AN, Brand PL (2015). Development and Validation of the Scan of Postgraduate Educational Environment Domains (SPEED): A Brief Instrument to Assess the Educational Environment in Postgraduate Medical Education. PloS One.

[51] Lutz G, Pankoke N, Goldblatt H, Hofmann M, Zupanic M (2017). Enhancing medical students' reflectivity in mentoring groups for professional development - a qualitative analysis. BMC Med Educ.

[52] Juenger J, Schultz JH, Schoenemann J, Wagener S, Drude N, Duelli R, Resch F (2009). AMEE Guide Supplements: Peer-assisted learning: A planning and implementation framework. Guide supplement 30.6--practical application. Med Teach.

[53] Gerlach D (2021). Was machen Mentor*innen im Vorbereitungsdienst?. Z Schul Professionsentw.

[54] Schaub-de Jong M (2013). Facilitating reflective learning: a PhD thesis report. Perspecti Med Educ.

[55] Hoos-Leistner H, Hoos-Leistner H (2019). Intrapersonelle und interpersonelle Kommunikation.

[56] Schmitz C, Trippolini M, Berchtold P (2021). Feedback-Kultur und psychologische Sicherheit. Schweiz Ärzteztg.

[57] Branch WT, Paranjape A (2002). Feedback and reflection: teaching methods for clinical settings. Acad Med.

[58] Lixenfeld C (2007). Wo Ärzte sprechen lernen. kma Gesundheitswiss.

[59] Homberg A, Hundertmark J, Krause J, Brunnée M, Neumann B, Loukanova S (2019). Promoting medical competencies through a didactic tutor qualification programme - a qualitative study based on the CanMEDS Physician Competency Framework. BMC Med Educ.

[60] Stenfors-Hayes T, Kalén S, Hult H, Dahlgren LO, Hindbeck H, Ponzer S (2010). Being a mentor for undergraduate medical students enhances personal and professional development. Med Teach.

[61] Franz HW, Kopp R (2003). Die Kollegiale Fallberatung: ein einfaches und effektives Verfahren zur 'Selbstberatung'. Sozialwiss Berufpraxis.

