# Supplementary material for: Professional Identity Formation in the model curriculum of human medicine in Oldenburg – a longitudinal approach
Source: GMS J Med Educ. 2026 Mar 23;43(3):Doc38. doi: 10.3205/zma001832 (PMC13054818; doi:10.3205/zma001832)
Supplement: Evaluation results 2012/13 – 2014/15 [file JME-43-38-s-001.pdf]

Attachment 1: Evaluation results 2012/13 – 2014/15

Semester	Cohort	Modul	RR<br>n<br>N		The assignments... are clearly formulated and easy to complete.	are clearly formulated.	actively involved the students in the teaching process.	are manageable in terms of content.	are meaningfully integrated into the modul.	motivate me to engage with scientific topics.	stimulate critical scientific engagement.
WiSe 12/13	A	M1.1	73%	M	2,52					2,93	2,48
			29	n	29					29	29
			40	SD	1,06					0,84	0,99
WiSe 12/13	A	M1.2	70%	M	2,96					3,63	3,44
			28	n	27					27	27
			40	SD	1,06					1,01	1,05
SuSe 13	A	M 1.3	55%	M	2,68					2,00	2,59
			22	n	22					22	22
			40	SD	0,95					0,82	1,05
SuSe 13	A	M 1.4	43%	M	3,41					3,71	3,29
			17	n	17					17	17
			40	SD	1,12					1,21	1,36
WiSe 13/14	A	M 2.1	44%	M		2,40	2,20			2,47	3,73
			15	n		15	15			15	15
			34	SD		0,63	0,41			0,64	0,80
WiSe 13/14	A	M 2.2	65%	M		2,55	2,73			4,00	3,59
			22	n		22	22			21	22
			34	SD		0,91	0,98			1,00	1,01
SuSe 14	A	M 2.3	62%	M		2,95		2,95	3,81		
			21	n		21		21	21		
			34	SD		1,28		1,02	1,25		
SuSe 14	A	M 2.4	35%	M		2,67		3,08	3,33		
			12	n		12		12	12		
			34	SD		1,07		1,38	1,15		
				Scale	a	a	a	a	a	a	a

Scale: a: (1 = “fully applies”; 5 = “does not apply at all”)

*Estimated value, as the number of participants was not archived.

WiSe = Winter Semester
SuSe = Summer Semester

Attachment 1 to Gehlhar K, Ankele M, Aits I, Dehlfing A, Ennen M. P*rofessional Identity Formation in the model curriculum of human medicine in Oldenburg – a longitudinal approach.* GMS J Med Educ. 2026;43(3):Doc38. DOI: 10.3205/zma001832

Semester	Cohort	Modul	RR<br>n<br>N		The assignments... help to understand the non-medical aspects of being a physician.	encourage me to analyze my own experiences.	The time required for the assignments is appropriate.	The series of sessions… encourages me to recognize my own strengths and weaknesses.	is helpful in preparing for my future professional role.	Concept: I am familiar with the concept and I know exactly what is expected of me.	I am familiar with the concept
WiSe 12/13	A	M1.1	73%	M						2,93	
			29	n						29	
			40	SD						0,84	
WiSe 12/13	A	M1.2	70%	M						3,11	
			28	n						27	
			40	SD						1,19	
SuSe 13	A	M 1.3	55%	M						2,50	
			22	n						22	
			40	SD						0,86	
SuSe 13	A	M 1.4	43%	M						3,59	
			17	n						17	
			40	SD						0,87	
WiSe 13/14	A	M 2.1	44%	M							1,64
			15	n							14
			34	SD							0,50
WiSe 13/14	A	M 2.2	65%	M							2,82
			22	n							22
			34	SD							0,91
SuSe 14	A	M 2.3	62%	M	3,52	3,30	3,76	3,62	3,57		3,05
			21	n	21	20	21	21	21		21
			34	SD	0,98	1,13	1,09	0,92	0,98		1,16
SuSe 14	A	M 2.4	35%	M	3,08	3,08	3,75	2,83	3,17		2,83
			12	n	12	12	12	12	12		12
			34	SD	1,24	1,24	1,22	1,27	1,40		1,34
				Scale	a	a	a	a	a	a	a

Scale: a: (1 = “fully applies”; 5 = “does not apply at all”)

Attachment 1 to Gehlhar K, Ankele M, Aits I, Dehlfing A, Ennen M. P*rofessional Identity Formation in the model curriculum of human medicine in Oldenburg – a longitudinal approach.* GMS J Med Educ. 2026;43(3):Doc38. DOI: 10.3205/zma001832

Semester	Cohort	Modul	RR<br>n<br>N		The coaching sessions …							
					are well structured and easy to follow in terms of content	offer a good balance between content volume and time	are well structured in terms of timing	are well integrated into the modul from a scheduling perspective	are meaningfully integrated into the modul	are well structured and allow for constructive work	gave me a good overview of the topic gave me a good overview of the topic	are well structured
WiSe 12/13	A	M1.1	73%	M	2,50	2,18			2,61	2,41	2,64	
			29	n	28	28			28	29	28	
			40	SD	0,88	0,77			1,03	0,68	0,91	
WiSe 12/13	A	M1.2	70%	M	2,78	2,59			3,30	2,78	3,26	
			28	n	27	27			27	27	27	
			40	SD	0,93	0,80			1,03	1,05	1,06	
SuSe 13	A	M 1.3	55%	M	2,50	3,00			2,36	2,68	2,43	
			22	n	22	22			22	22	21	
			40	SD	0,86	0,76			0,95	0,89	1,03	
SuSe 13	A	M 1.4	43%	M	3,06	3,00			3,41	3,00	3,19	
			17	n	17	17			17	17	16	
			40	SD	0,97	1,12			1,28	1,06	1,17	
WiSe 13/14	A	M 2.1	44%	M	3,27	2,21			2,29			2,87
			15	n	15	14			14			15
			34	SD	0,80	0,80			0,91			1,25
WiSe 13/14	A	M 2.2	65%	M	2,41	2,55			3,32			1,95
			22	n	22	22			22			22
			34	SD	0,91	1,01			1,13			0,72
SuSe 14	A	M 2.3	62%	M	2,67	3,00	2,67	3,81				
			21	n	21	21	21	21				
			34	SD	0,97	1,41	1,02	1,03				
SuSe 14	A	M 2.4	35%	M	2,50	2,58	2,33	3,75				
			12	n	12	12	12	12				
			34	SD	1,17	1,16	1,07	0,87				
				Scale	a	a	a	a	a	a	a	a

Scale: a: (1 = “fully applies”; 5 = “does not apply at all”)

*Estimated value, as the number of participants was not archived.

Semester	Cohort	Modul	RR<br>n<br>N		The coaching sessions …			How do you rate …				
					enable constructive work	The structure of the coaching sessions is understandable to me	Overall, I liked the coaching sessions	the overall concept/ PIF session series?	the atmosphere in the group?	the outcomes of the sessions (learning goals & content)?	your coaches?	the coaching sessions overall?
			73%	M		2,52	2,25	2,28	1,79	2,36	1,96	
			29	n		29	28	29	29	28	28	
WiSe 12/13	A	M1.1	40	SD		0,78	0,65	0,84	0,68	0,87	0,69	
			70%	M		3,04	3,19	2,96	2,00	3,22	2,26	
			28	n		27	27	25	27	27	27	
WiSe 12/13	A	M1.2	40	SD		1,06	0,92	1,06	0,68	1,05	0,86	
			55%	M		2,41	2,68	2,77	3,77	2,73	3,77	
			22	n		22	22	22	22	22	22	
SuSe 13	A	M 1.3	40	SD		0,80	1,04	1,23	0,87	1,03	0,92	
			43%	M		3,29	2,94	3,00	2,13	2,60	2,12	
			17	n		17	17	16	16	15	17	
SuSe 13	A	M 1.4	40	SD		0,92	0,83	1,10	0,89	1,12	0,93	
			44%	M	2,20	2,47		3,64	2,93	1,86	2,57	2,14
			15	n	15	15		14	14	14	14	14
WiSe 13/14	A	M 2.1	34	SD	0,56	0,64		1,01	1,07	0,53	0,65	0,86
			65%	M	2,43	2,41		2,81	2,18	2,73	2,14	2,68
			22	n	21	22		21	22	22	22	22
WiSe 13/14	A	M 2.2	34	SD	1,08	0,85		1,03	1,10	1,16	0,83	0,95
			62%	M				3,05	2,52		2,19	
			21	n				21	21		21	
SuSe 14	A	M 2.3	34	SD				1,16	0,98		1,17	
			35%	M				2,75	2,58		1,83	
			12	n				12	12		12	
SuSe 14	A	M 2.4	34	SD				1,42	0,79		0,72	
				Scale	a	a	a	b	b	b	b	b

Scale: a: (1 = “fully applies”; 5 = “does not apply at all”)

Semester	Cohort	Modul	RR<br>n<br>N		The assignments... are clearly formulated and easy to complete.	are clearly formulated.	actively involved the students in the teaching process.	are manageable in terms of content.	are meaning-fully integrated into the module.	motivate me to engage with scientific topics.	stimulate critical scientific engagement.
WiSe 14/15	A	M 3.1	30%	M		3,25		3,27	3,67		
			12	n		12		11	12		
			*40**	SD		1,36		1,35	0,89		
WiSe 13/14	B	M1.1	53%	M		2,67	2,90			3,71	3,05
			21	n							
			*40**	SD		1,11	1,04			1,10	1,28
WiSe 13/14	B	M1.2	43%	M		2,41	2,65			3,94	3,12
			17	n							
			*40**	SD		0,94	0,93			1,09	1,36
SuSe 14	B	M 1.3	43%	M		2,59		3,06	3,06		
			17	n		17		17	17		
			*40**	SD		1,00		0,90	1,03		
SuSe 14	B	M 1.4	28%	M		2,36		2,36	3,10		
			11	n		11		11	10		
			*40**	SD		0,67		0,67	0,99		
				Scale	a	a	a	a	a	a	a

Scale: a: (1 = “fully applies”; 5 = “does not apply at all”)

*Estimated value, as the number of participants was not archived.

Semester	Cohort	Modul	RR<br>n<br>N		**The assignments...** help to understand the non-medical aspects of being a physician.	**The assignments...** encourage me to analyze my own experiences.	**The assignments...** The time required for the assignments is appropriate.	**The series of sessions…** encourages me to recognize my own strengths and weaknesses.	**The series of sessions…** is helpful in preparing for my future professional role.	**Concept** I am familiar with the concept
WiSe 14/15	A	M 3.1	30%	M	3,27	3,18	3,55	3,33	3,50	2,83
			12	n	11	11	11	12	12	12
			*40**	SD	1,19	1,17	1,21	1,15	1,09	1,11
WiSe 13/14	B	M1.1	53%	M						2,14
			21	n						21
			*40**	SD						0,91
WiSe 13/14	B	M1.2	43%	M						2,47
			17	n						17
			*40**	SD						1,12
SuSe 14	B	M 1.3	43%	M	3,12	2,88	3,53	3,18	3,47	3,18
			17	n	17	17	17	17	15	17
			*40**	SD	0,99	1,05	1,12	1,24	1,06	1,19
SuSe 14	B	M 1.4	28%	M	2,80	2,40	3,27	2,82	2,70	2,18
			11	n	10	10	11	11	10	11
			*40**	SD	1,03	0,84	0,79	1,08	1,16	0,87
				Scale	a	a	a	a	a	a

Scale: a: (1 = “fully applies”; 5 = “does not apply at all”)

*Estimated value, as the number of participants was not archived.

Semester	Cohort	Modul	RR<br>n<br>N		The coaching sessions …							
					are well structured and easy to follow in terms of content	offer a good balance between content volume and time	are well structured in terms of timing	are well integrated into the module from a scheduling perspective	are meaningfully integrated into the module	are well structured	enable constructive work	The structure of the coaching sessions is under-standable to me
WiSe 14/15	A	M 3.1	30%	M	2,92	3,09	3,08	3,67				
			12	n	12	11	12	12				
			*40**	SD	1,16	1,04	1,31	1,30				
WiSe 13/14	B	M1.1	53%	M	2,10	2,86			3,19	2,10	2,43	2,19
			21	n								
			*40**	SD	0,77	1,06			1,25	0,94	0,87	0,93
WiSe 13/14	B	M1.2	43%	M	2,53	2,82			3,44	2,53	2,71	2,44
			17	n								
			*40**	SD	0,87	0,95			0,73	0,80	1,10	0,73
SuSe 14	B	M 1.3	43%	M	3,18	2,82	3,12	3,65				
			17	n	17	17	17	17				
			*40**	SD	1,24	1,13	0,93	1,17				
SuSe 14	B	M 1.4	28%	M	2,36	2,55	2,82	3,18				
			11	n	11	11	11	11				
			*40**	SD	0,92	1,21	1,25	1,08				
				Scale	a	a	a	a	a	a	a	a

Scale: a: (1 = “fully applies”; 5 = “does not apply at all”)

*Estimated value, as the number of participants was not archived.

Semester	Cohort	Modul	RR<br>n<br>N		How do you rate… the overall concept/PIF session series?	the atmosphere in the group?	the outcomes of the sessions (learning goals & content)?	your coaches?	the coaching sessions overall?
			30%	M	3,17	2,00		2,17	
			12	n	12	12		12	
WiSe 14/15	A	M 3.1	*40**	SD	1,19	0,60		1,11	
			53%	M	2,24	1,86	2,25	1,95	2,29
			21	n					
WiSe 13/14	B	M1.1	*40**	SD	1,04	1,06	1,07	1,02	1,23
			43%	M	2,71	1,65	2,59	2,29	
			17	n					
WiSe 13/14	B	M1.2	*40**	SD	1,05	0,93	0,94	0,92	
			43%	M	3,00	2,35		2,47	
			17	n	16	17		17	
SuSe 14	B	M 1.3	*40**	SD	1,03	0,86		0,87	
			28%	M	2,27	1,73		2,00	
			11	n	11	11		11	
SuSe 14	B	M 1.4	*40**	SD	1,01	0,65		0,89	
				Scale	b	b	b	b	b

Scale: b (1 = "sehr gut"; 5 = "ungenügend")

*Schätzung, da Teilnahmezahl nicht archiviert.

Attachment 1 to Gehlhar K, Ankele M, Aits I, Dehlfing A, Ennen M. P*rofessional Identity Formation in the model curriculum of human medicine in Oldenburg – a longitudinal approach.* GMS J Med Educ. 2026;43(3):Doc38. DOI: 10.3205/zma001832
