# Supplementary material for: Professional Identity Formation in the model curriculum of human medicine in Oldenburg – a longitudinal approach
Source: GMS J Med Educ. 2026 Mar 23;43(3):Doc38. doi: 10.3205/zma001832 (PMC13054818; doi:10.3205/zma001832)
Supplement: Survey of study years 1 and 1-4 (2021 and 2023) [file JME-43-38-s-002.pdf]

Attachment 2: Survey of study years 1 and 1-4 (2021 and 2023)

				The PIF-Curriculum …							
**Semester**	**Study year**	**RR n N**		3.1 was announced in a timely manner	3.2 was clearly structured	3.3 was well organized	3 3.4 had an appropriate group size for the session format	3.5 had a reasonable balance between content volume and time	3.6 had an appropriate method of performance assessment	3.7 clearly stated learning objectives	3.8 was appropriately adapted to the organizational challenges of the COVID-19 pandemic
SuSe 2021 (virtual)	1	9%	M	1,64	2,73	2,82	1,36	1,36	1,73	3,10	2,36
		11	n	11	11	11	11	11	11	10	11
		120	SD	0,924	0,786	0,603	0,505	0,505	0,647	0,738	0,809
SuSe 2023	1	17%	M								
		20	n								
		117	SD								
SuSe 2023	2	12%	M								
		11	n								
		91	SD								
SuSe 2023	3	7%	M								
		6	n								
		82	SD								
SuSe 2023	1 - 4	11%	M								
		37	n								
		354	SD								
			Scale	a	a	a	a	a	a	a	a

Scale: a (1 = “applies”, 2 = “somewhat applies”, 3 = “rather does not apply”, 4 = “does not apply”)

Attachment 2 to Gehlhar K, Ankele M, Aits I, Dehlfing A, Ennen M. P*rofessional Identity Formation in the model curriculum of human medicine in Oldenburg – a longitudinal approach.* GMS J Med Educ. 2026;43(3):Doc38. DOI: 10.3205/zma001832

Semester	Study year	RR<br>n<br>N		Organization: 3.1 The portfolio is an appropriate form of performance assessment.	Organization: 3.2 I am familiar with the learning objectives of the PIF curriculum	Organization: 3.3 The content volume is appropriate in relation to the time available	The PIF-Curriculum…: 5.1 fits well into the overall curriculum.	The PIF-Curriculum…: 4.1 covered relevant topics.	The PIF-Curriculum…: 4.2 was helpful for my personal professional development.	The PIF-Curriculum…: 4.3 provides a meaningful supplement to other curricular areas and content.
SuSe 2021 (virtual)	1	9%	M					2,18	2,45	2,45
		11	n					11	11	11
		120	SD					0,751	0,934	0,820
SuSe 2023	1	17%	M	1,85	2,25	1,90	1,93			
		20	n	20	20	20	14			
		117	SD	0,875	0,967	0,308	0,829			
SuSe 2023	2	12%	M	2,55	2,18	2,00	2,29			
		11	n	11	11	11	7			
		91	SD	1,036	0,874	0,447	0,756			
SuSe 2023	3	7%	M	2,40	2,00	2,20	2,00			
		6	n	5	5	5	4			
		82	SD	0,548	1,000	0,447	0,816			
SuSe 2023	1 - 4	11%	M	2,16	2,19	1,97	2,08			
		37	n	37	37	37	26			
		354	SD	0,928	0,908	0,372	0,796			
			Scale	a	a	b	a	a	a	a

Scale: a (1 = “applies”, 2 = “somewhat applies”, 3 = “rather does not apply”, 4 = “does not apply”)

b (1 = “too little”, 2 = “appropriate”, 3 = “too much”)

				**The PIF-curriculum…**		**The tutor …**						
**Semester**	**Study year**	**RR**<br>**n**<br>**N**		4.4 helped me prepare for the observer-ship(s).	4.5 helped me reflect on the observer-ship(s) afterwards.	5.1 appeared well prepared.	5.2 structured the session clearly.	5.3 was very committed to delivering the course content.	5.4 explained things clearly.	5.5 appeared competent in the subject matter.	5.6 encouraged my independent study.	5.7 was available to answer questions.
SuSe 2021 (virtual)	1	9%	M	2,73	2,82	2,82	2,91	2,55	2,55	2,45	2,36	1,70
		11	n	11	11	11	11	11	11	11	11	10
		120	SD	1,009	1,079	1,079	0,944	1,036	1,036	1,036	0,924	0,823
SuSe 2023	1	17%	M	2,29	1,93	2,00	1,87		1,80	1,47		2,00
		20	n	14	14	15	15		15	15		15
		117	SD	0,994	0,997	1,069	0,915		0,941	0,834		1,069
SuSe 2023	2	12%	M	3,00	2,57	1,86	2,14		2,00	1,43		2,43
		11	n	7	7	7	7		7	7		7
		91	SD	1,000	0,787	0,900	1,215		1,155	0,787		1,134
SuSe 2023	3	7%	M	3,00	2,25	1,50	1,75		1,50	1,25		1,50
		6	n	4	4	4	4		4	4		4
		82	SD	0,816	1,258	0,577	0,957		0,577	0,500		1,000
SuSe 2023	1 - 4	11%	M	2,65	2,12	1,93	1,96		1,81	1,41		2,07
		37	n	26	26	27	27		27	27		27
		354	SD	1,018	0,993	0,958	0,980		0,921	0,747		1,072
			Scale	a	a	a	a	a	a	a	a	a

Scale: a (1 = “applies”, 2 = “somewhat applies”, 3 = “rather does not apply”, 4 = “does not apply”)

Attachment 2 to Gehlhar K, Ankele M, Aits I, Dehlfing A, Ennen M. P*rofessional Identity Formation in the model curriculum of human medicine in Oldenburg – a longitudinal approach.* GMS J Med Educ. 2026;43(3):Doc38. DOI: 10.3205/zma001832

				The tutor …					Working in the small group …		
**Semester**	**Study year**	**RR n N**		5.8 clarified the learning objectives to be achieved.	5.9 made assessment criteria transparent (if applicable).	5.10 adhered to the scheduled time frame.	5.11 supported my professional development well, particularly due to their own experience as a physician.	5.12 created a working atmosphere that allowed for deeper/ personal questions.	5.13 provided a protected/ confidential space for discussion.	5.14 was marked by respectful interaction.	5.15 offered sufficient space for reflection.
SuSe 2021 (virtual)	1	9%	M	2,91	2,45	1,36	2,45	2,82	2,00	1,73	2,18
		11	n	11	11	11	11	11	11	11	11
		120	SD	0,944	0,934	0,674	0,688	1,079	1,000	0,905	0,751
SuSe 2023	1	17%	M	1,53				1,47	1,27	1,33	1,53
		20	n	15				15	15	15	15
		117	SD	0,915				0,915	0,799	0,816	0,915
SuSe 2023	2	12%	M	1,57				2,00	1,86	1,86	1,86
		11	n	7				7	7	7	7
		91	SD	0,535				1,155	1,069	1,069	0,900
SuSe 2023	3	7%	M	1,00				1,50	1,25	1,25	1,25
		6	n	4				4	4	4	4
		82	SD	0,000				1,000	0,500	0,500	0,500
SuSe 2023	1 - 4	11%	M	1,44				1,63	1,41	1,44	1,56
		37	n	27				27	27	27	27
		354	SD	0,751				0,967	0,844	0,847	0,847
			Scale	a	a	a	a	a	a	a	a

Scale: a (1 = “applies”, 2 = “somewhat applies”, 3 = “rather does not apply”, 4 = “does not apply”)

Semester	Study year	RR<br>n<br>N		Working in the small group … 5.16 took place on a regular basis.	Working in the small group … 5.17 provided a suitable setting for PIF.	6.1 The topics addressed also offer meaningful points of connection beyond the PIF curriculum.
SuSe 2021 (virtual)	1	9%	M	1,73	1,64	2,80
		11	n	11	11	10
		120	SD	0,905	0,505	0,789
SuSe 2023	1	17%	M			2,53
		20	n			15
		117	SD			0,990
SuSe 2023	2	12%	M			3,57
		11	n			7
		91	SD			0,787
SuSe 2023	3	7%	M			3,00
		6	n			4
		82	SD			0,816
SuSe 2023	1 - 4	11%	M			2,93
		37	n			27
		354	SD			0,997
			Scale	a	a	b

Scale: a (1 = “applies”, 2 = “somewhat applies”, 3 = “rather does not apply”, 4 = “does not apply”)
b (1 = “always”, 2 = “often”, 3 = “occasionally”, 4 = “rarely”, 5 = “never”)
