# Supplementary material for: Professional Identity Formation in the model curriculum of human medicine in Oldenburg – a longitudinal approach
Source: GMS J Med Educ. 2026 Mar 23;43(3):Doc38. doi: 10.3205/zma001832 (PMC13054818; doi:10.3205/zma001832)
Supplement: Survey on PIF year 5 in 2021 and 2023 [file JME-43-38-s-003.pdf]

Attachment 3: Survey on PIF year 5 in 2021 and 2023

				B. Assessment of the concept			C.4 Evaluation of the content/ tasks in the PIF year 5 handbook		
**Semester**	**Study year**	**RR n N**		B.1 The longitudinal PIF track from the previous study years is coherently continued in year 5.	B.2 The preceding PIF curriculum prepared me well for PIF year 5.	B.3 The content offered in the PIF year 5 sessions aligns with my interests in this phase of study.	C.1 The handbook was helpful for my learning progress during medical school.	C.2 The task descriptions in the handbook were clearly formulated.	C.3 The handbook was helpful in organizing and planning my fifth year of study.
SuSe 2021	5	22%	M	3,54	3,17	3,00	2,64	3,75	2,91
		13	n	13	12	12	11	12	11
		*60**	SD	0,660	1,030	0,739	0,674	0,452	0,944
SuSe 2023	5	22%	M	3,15	2,58	3,23	2,20	3,20	2,10
		13	n	13	12	13	10	10	10
		*60**	SD	0,899	1,084	0,725	1,033	0,789	0,876
			Scale:	a	a	a	a	a	a

Scale: a (1 = “does not apply”, 4 = “applies”)
b (1 = “not helpful”, 2 = “rather not helpful”, 3 = “rather helpful”, 4 = “helpful”)

*Estimated value, as the number of participants was not archived.

Semester	Study year	RR<br>n<br>N		C.4.1 General information/ guidance for Year 5	C.4.2 Self-assessment of competency development during medical school	C.4.3 Creating a CV	C.4.4 Study planning for year 5	C.4.5 Evidence-based clinical decision-making (PICO)	C.4.6 Reflection on the research project	C.4.7 Career perspective
				**C.4 Evaluation of the content/tasks in the PIF year 5 handbook**						
SuSe 2021	5	22%	M	2,83	2,67	3,09	3,33	2,67	3,25	3,25
		13	n	12	12	11	12	12	12	12
		*60**	SD	0,718	0,778	1,221	0,888	0,492	0,452	0,754
SuSe 2023	5	22%	M	3,00	2,55	2,00	2,55	1,64	2,18	2,82
		13	n	10	11	11	11	11	11	11
		*60**	SD	0,667	1,036	1,000	0,934	0,674	1,079	1,079
			Scale:	b	b	b	b	b	b	b

Semester	Study year	RR<br>n<br>N		E.1 How many meetings with your mentor took place over the course of the year?	My Mentor..<br>E.2 supported me in planning and preparing the sessions.	E.3 made sufficient time available for the sessions.	E.4 was familiar with the handbook.	E.5 took their role seriously.	E.6 created a pleasant atmosphere.	E.7 supported me with my concerns.	E.8 provided good guidance
SuSe 2021	5	22%	M	3,25	3,45	3,91	3,64	3,91	3,91	3,91	3,91
		13	n	12	11	11	11	11	11	11	11
		*60**	SD	0,622	0,688	0,302	0,505	0,302	0,302	0,302	0,302
SuSe 2023	5	22%	M	3,00	3,56	3,90	3,50	3,80	4,00	3,80	4,00
		13	n	10	9	10	10	10	10	10	10
		*60**	SD	0,667	0,527	0,316	0,707	0,422	0,000	0,422	0,000
			Scale:	c	a	a	a	a	a	a	a

Semester	Study year	RR<br>n<br>N		My Mentor..<br>E.9 serves as a role model for my professional development	E.10 serves as a role model for my personal development	E Mentoring-Program<br>E.11 The mentoring sessions helped me identify a topic and/or super-visor for my research project.	E.12 The mentoring sessions encouraged me to reflect on my professional goals.	E.13 The mentoring sessions made me aware of the need for personal initiative.	E.14 The mentoring sessions gave me a motivational boost for the remainder of my studies.	E.15 I am satisfied with the feedback I received.	E.16 I learned a lot from the feedback I received.
SuSe 2021	5	22%	M	3,82	3,36	1,25	3,36	3,18	3,36	3,82	3,08
		13	n	11	11	8	11	11	11	11	12
		*60**	SD	0,405	0,809	0,707	1,027	1,168	0,809	0,405	0,669
SuSe 2023	5	22%	M	3,67	3,60	1,13	3,00	2,67	2,89	3,88	3,00
		13	n	9	10	8	9	9	9	8	7
		*60**	SD	0,500	0,516	0,354	1,000	1,000	1,167	0,354	1,155
			Scale:	a	a	a	a	a	a	a	a

Scale: a (1 = “does not apply”, 4 = “applies”)
c (1 = "none", 2 = "1 -3", 3 = "4", 4 = 5 - 8", 5 = ">8")

*Estimated value, as the number of participants was not archived.

Attachment 3 to Gehlhar K, Ankele M, Aits I, Dehlfing A, Ennen M. P*rofessional Identity Formation in the model curriculum of human medicine in Oldenburg – a longitudinal approach.* GMS J Med Educ. 2026;43(3):Doc38. DOI: 10.3205/zma001832

Semester	Study year	RR n N		E Mentoring-Program: E.17 Mentoring was very valuable to me in completing year 5.	E.18 I prepared for and/or followed up on the mentoring sessions.	E.19 I kept to agreements made with my mentor.	E.20 I kept my mentor informed about the progress of my academic performance.	E.21 Overall, I rate the mentoring program with the following school grade (1–5).	F. Workshop-Program: F.1 How many workshops from the PIF year 5 series did you attend?	F.2 I am very satisfied with the thematic selection of the workshops.	F.3 I am very satisfied with the scheduling of the workshops I attended.
SuSe 2021	5	22%	M	2,92	4,33	4,75	4,58	1,75	4,83	3,17	3,17
		13	n	12	12	12	12	12	12	12	12
		*60**	SD	0,996	0,888	0,452	0,669	0,754	0,718	0,718	0,835
SuSe 2023	5	22%	M	2,67	3,50	4,80	4,60	2,80	4,60	3,50	3,20
		13	n	9	10	10	10	10	10	10	10
		*60**	SD	1,000	1,269	0,422	0,516	1,619	0,843	0,527	0,422
			Scale:	a	a	a	a	d	e	a	a

Semester	Study year	RR n N		F. Workshop-Program: F.4 I am very satisfied with the content organization of the workshops I attended.	F.5 The workshop format motivates me to engage more deeply with the course content.	F.6 I prepared for and/or followed up on the workshop content.	F.8 The PIF year 5 workshop series is thematically well structured.	F.9 The PIF year 5 workshops complement the sessions from previous study years appropriately.	F.10 Overall, I find the concept of the PIF year 5 workshop series to be good.
SuSe 2021	5	22%	M	3,58	3,00	2,75	4,17	4,18	4,42
		13	n	12	12	12	12	11	12
		*60**	SD	0,515	0,739	0,866	0,718	0,751	0,515
SuSe 2023	5	22%	M	3,20	2,70	2,20	4,20	3,80	4,40
		13	n	10	10	10	10	10	10
		*60**	SD	0,632	0,675	0,632	0,632	1,549	0,699
			Scale:	a	a	a	f	f	f

Scale:
a (1 = “does not apply”, 4 = “applies”)
f (1 = “does not apply at all”, 5 = “fully applies”)
d (school grades: 1 = excellent; 5 = poor)

*Estimated value, as the number of participants was not archived.

Semester	Study year	RR n N		G. Self-assessment of one's own competence and attitudes toward professional development								
				G.1 I find it easy to manage tasks in clinical work.	G.2 I feel competent in dealing with patients.	G.3 I am aware of my scope of action in patient care during clinical practice.	G.4 I find it easy to interpret others' reactions to my behavior.	G.5 When I make a mistake, I know appropriate strategies to deal with it.	G.6 I feel competent in interacting with colleagues.	G.7 I find it easy to admit to colleagues when I've failed to do something.	G.8 If I notice a mistake by a colleague, I address it.	G.9 I find feedback from colleagues helpful.
SuSe 2021	5	22%	M	3,00	3,18	3,18	3,09	3,18	2,91	3,27	2,55	3,82
		13	n	11	11	11	11	11	11	11	11	11
		*60**	SD	0,775	0,751	0,603	0,302	0,603	0,539	0,905	0,688	0,405
SuSe 2023	5	22%	M	3,20	3,50	3,22	3,10	3,00	3,30	3,60	2,40	3,60
		13	n	10	10	9	10	10	10	10	10	10
		*60**	SD	0,919	0,527	0,667	0,568	0,471	0,675	0,699	0,699	0,516
		Scale:		a	a	a	a	a	a	a	a	a

Semester	Study year	RR n N		G. Self-assessment of one's own competence and attitudes toward professional development								
				G.10 I recognize situations where I need a second opinion.	G.11 If a patient asks me about my level of training, I answer honestly.	G.12 I recognize when I'm having a bad day in the clinic.	G.13 I am able to leave my private problems at home.	G.14 Experiences in clinical work are sometimes difficult to cope with.	G.15 During my studies, I developed strategies to deal with clinical experiences.	G.16 A physician serves as a role model in society.	G.17 It is important for a physician to stay up to date with current treatment options.	G.18 It is important for a physician to incorporate current research findings into clinical work.
SuSe 2021	5	22%	M	3,36	4,00	3,45	3,27	2,00	2,91	3,00	3,73	3,73
		13	n	11	11	11	11	11	11	11	11	11
		*60**	SD	0,674	0,000	0,934	0,786	0,894	0,701	0,447	0,467	0,467
SuSe 2023	5	22%	M	3,50	3,80	3,40	3,30	2,50	3,33	3,10	3,90	3,30
		13	n	10	10	10	10	10	9	10	10	10
		*60**	SD	0,527	0,422	0,516	0,675	0,972	0,707	0,876	0,316	0,823
		Scale:		a	a	a	a	a	a	a	a	a

Scale: a (1 = "does not apply", 4 = "applies") *Estimated value, as the number of participants was not archived.
