# Supplementary material for: Professional Identity Formation in the model curriculum of human medicine in Oldenburg – a longitudinal approach
Source: GMS J Med Educ. 2026 Mar 23;43(3):Doc38. doi: 10.3205/zma001832 (PMC13054818; doi:10.3205/zma001832)
Supplement: Evaluation of PIF 1-5 (2024) [file JME-43-38-s-004.pdf]

Attachment 4: Evaluation of PIF 1–5 (2024)

			2. Organization			4. Alignment with the curriculum PIF			
**Study year**	**RR n N**		2.1 The portfolio is an appropriate form of performance assessment.	2.2 I am familiar with the learning objectives of the PIF curriculum.	2.3 The amount of content in relation to time is…	4.1 …is well integrated into the overall curriculum.	4.2 …helped me prepare for the observership(s) or block internship(s).	4.3 …helped me reflect on the observership(s) or block internship(s) afterwards.	9.1 The topics addressed provide meaningful points of connection for my professional development beyond the PIF curriculum.
1	42%	M	2,06	2,27	2,02	2,13	2,46	2,34	2,87
	49	n	49	49	46	38	39	38	38
	117	SD	0,876	0,811	0,333	0,704	1,047	1,021	0,875
2	51%	M	2,11	2,35	2,08	2,20	2,89	2,51	3,02
	64	n	62	62	62	54	55	55	55
	125	SD	0,889	0,832	0,329	0,786	0,936	0,879	0,952
3	53%	M	2,44	2,38	2,05	2,35	3,33	3,00	3,22
	46	n	45	45	44	40	36	37	37
	86	SD	0,785	1,029	0,429	0,700	0,793	0,943	0,854
4	36%	M	2,42	2,00	2,10	2,23	2,81	2,11	2,74
	31	n	31	31	29	26	27	27	27
	87	SD	0,848	0,894	0,310	0,710	0,921	1,155	1,130
5	20%	M	2,73	2,00	2,09	2,00	2,33	1,75	2,33
	12	n	11	11	11	12	12	12	12
	59	SD	0,905	0,447	0,302	0,853	0,888	0,866	0,651
Total	43%	M	2,26	2,26	2,06	2,21	2,83	2,46	2,93
	202	n	198	198	192	170	169	169	169
	474	SD	0,872	0,874	0,349	0,739	0,974	1,024	0,946
		Scale	a	a	b	a	a	a	e

Scale: a (1 = “applies”, 2 = “somewhat applies”, 3 = “rather does not apply”, 4 = “does not apply”)
b (1 = “too little”, 2 = “appropriate”, 3 = “too much”)
e (1 = "always", 2 = "often", 3 = "sometimes", 4 = "rarely", 5 = "never")

Attachment 4 to Gehlhar K, Ankele M, Aits I, Dehlfing A, Ennen M. P*rofessional Identity Formation in the model curriculum of human medicine in Oldenburg – a longitudinal approach.* GMS J Med Educ. 2026;43(3):Doc38. DOI: 10.3205/zma001832

Study year	RR<br>n<br>N		5. Design of the sessions<br>5.1 The session was clearly structured.	5.2 The learning objectives were addressed.	5.3 There was opportunity to ask questions.	5 4 The atmosphere encouraged open discussion, including on personal issues.	5.5 The tutor structured the session according to the PIF handbook.	5.6 The tutor appeared competent in the subject matter.	5.7 The time allocated for the session content was…
1	42%	M	2,28	1,89	1,41	1,62	1,69	1,74	2,16
	49	n	39	38	39	39	39	38	37
	117	SD	1,025	0,798	0,818	1,016	0,832	0,891	0,442
2	51%	M	2,23	2,07	1,42	1,85	1,95	1,76	2,21
	64	n	56	54	55	55	55	54	53
	125	SD	0,874	0,843	0,762	1,008	1,026	1,027	0,495
3	53%	M	2,18	2,10	1,38	1,60	1,90	1,74	2,31
	46	n	40	39	40	40	39	38	35
	86	SD	0,813	0,754	0,774	0,900	0,821	0,828	0,471
4	36%	M	1,96	2,00	1,22	1,48	1,73	1,37	2,20
	31	n	27	26	27	27	26	27	25
	87	SD	0,854	0,748	0,506	0,935	0,874	0,792	0,408
5	20%	M	1,92	1,83	1,25	1,42	1,70	1,25	2,17
	12	n	12	12	12	12	10	12	12
	59	SD	0,515	0,389	0,452	0,669	0,949	0,452	0,389
Total	43%	M	2,17	2,01	1,36	1,65	1,83	1,65	2,22
	202	n	174	169	173	173	169	169	162
	474	SD	0,874	0,771	0,724	0,956	0,906	0,894	0,456
		Scale	a	a	a	a	a	a	c

Scale: a (1 = “applies”, 2 = “somewhat applies”, 3 = “rather does not apply”, 4 = “does not apply”)
c (1 = “too short”, 2 = “appropriate”, 3 = “too long”)

Study year	RR<br>n<br>N		6. Group work …<br>6.1 …provided a safe and confidential space for exchange.	6.2 …was characterized by respectful interaction.	6.3 …allowed sufficient room for reflection.	6.4 The group size was…	7. Evaluation of the handbook<br>7.1 The handbook was helpful for my learning progress in medical school.	7.2 The task descriptions in the handbook were clearly formulated.	7 3 The handbook content fit well into this year of study.
1	42%	M	1,67	1,54	1,51	2,10	2,94	2,32	2,32
	49	n	39	39	39	39	36	37	34
	117	SD	0,927	0,913	0,823	0,307	0,860	0,784	0,843
2	51%	M	1,65	1,45	1,73	2,07	2,86	2,50	2,53
	64	n	54	55	55	55	50	52	51
	125	SD	0,935	0,812	0,891	0,262	0,881	0,960	0,857
3	53%	M	1,62	1,26	1,64	2,08	3,11	2,27	2,86
	46	n	37	35	36	37	37	37	35
	86	SD	0,861	0,561	0,867	0,363	0,875	0,838	0,810
4	36%	M	1,52	1,15	1,41	2,07	2,96	2,17	2,52
	31	n	27	27	27	27	27	24	23
	87	SD	0,802	0,456	0,636	0,267	0,808	0,816	0,947
5	20%	M	1,50	1,33	1,42	2,08	2,58	2,17	2,42
	12	n	12	12	12	12	12	12	12
	59	SD	0,798	0,492	0,669	0,289	0,900	0,718	0,793
Total	43%	M	1,62	1,38	1,59	2,08	2,93	2,33	2,55
	202	n	169	168	169	170	162	162	155
	474	SD	0,880	0,732	0,820	0,296	0,864	0,856	0,862
		Scale	a	a	a	d	a	a	a

Scale: a (1 = “applies”, 2 = “somewhat applies”, 3 = “rather does not apply”, 4 = “does not apply”)
d (1 = “too small”, 2 = “appropriate”, 3 = “too large”)

Study year	RR<br>n<br>N		8. Workshop program (Year 5)<br>8.2 I am very satisfied with the thematic selection of the workshop series.	8.3 I am very satisfied with the scheduling of the workshops I attended.	8.4 I am very satisfied with the content structure of the workshops I attended.	8.5 The instructors…<br>8.5.1 appeared professionally competent.	8.5.2 explained clearly.	8.5.3 appeared well prepared.	8.5.4 actively involved students in the learning process.	8.5.5 motivated me to engage more deeply with the topic.
5	20%	M	2,00	1,90	2,10	1,50	1,70	1,60	1,70	2,00
	12	n	10	10	10	10	10	10	10	10
	59	SD	0,667	0,738	0,876	0,707	0,823	0,843	0,823	0,816
		Scale	a	a	a	a	a	a	a	a

Scale: a (1 = “applies”, 2 = “somewhat applies”, 3 = “rather does not apply”, 4 = “does not apply”)

Study year	RR<br>n<br>N		8 6 The year 5 PIF workshops appropriately complement the sessions from previous study years.	8.7 Overall, I find the concept of the Year 5 PIF workshop series to be good.
5	20%	M	1,90	1,40
	12	n	10	10
	59	SD	0,876	0,516
		Scale	a	a
