# Supplementary material for: Professional Identity Formation in the model curriculum of human medicine in Oldenburg – a longitudinal approach
Source: GMS J Med Educ. 2026 Mar 23;43(3):Doc38. doi: 10.3205/zma001832 (PMC13054818; doi:10.3205/zma001832)
Supplement: Current questionnaire [file JME-43-38-s-005.pdf]

Attachment 5: Current questionnaire

Attachment 5 to Gehlhar K, Ankele M, Aits I, Dehlfing A, Ennen M. P*rofessional Identity Formation in the model curriculum of human medicine in Oldenburg – a longitudinal approach.* GMS J Med Educ. 2026;43(3):Doc38. DOI: 10.3205/zma001832

Attachment 5: Current questionnaire

Questionnaire

This sheet will be processed by a computer. Please, mark your answers using the following scheme: ○⊗○
If you want to invalidate an answer, use the following mark: ○●⊗.
Digits should look like this: 0 1 2 3 4 5 6 7 8 9; and corrections like this:

Dear students,<br><br>We would be very grateful if you could give us feedback on your experiences with the Professional Identity Formation (PIF) track during the current academic year.<br><br>Kind regards<br><br>Michael Ankele<br>Evaluation Coordinator for the Medical Program<br><br>For questions: evaluation.medizin@uol.de

<[B]DATENSCHUTZERKLÄRUNG[/B]>	○ I consent to the processing of my personal data as explained at <link>.

PREVIEW
Do not fill in!

1. General Information

We would first like to ask for some information about yourself.

1.1 How old are you?	○ 17-21 ○ 22-26 ○ >26 ○ n.a.
1.2 What is your gender?	○ male ○ female<br>○ diverse ○ n.a
1.3 Do you have a prior vocational qualification in a health profession?	○ yes ○ no<br>○ <K.A.>
1.4 In which year of study are you currently taking part in PIF?	○ 1 ○ 2<br>○ 3 ○ 4<br>○ 6 ○ <K.A.>

2. Organization

How do you assess the organizational framework of the PIF sessions this year?

	applies	somewhat applies	rather does not apply	does not apply	n.a.
2.1 The portfolio is an appropriate form of performance assessment.	○	○	○	○	○
2.2 I am familiar with the learning objectives of the PIF curriculum.	○	○	○	○	○

	too low	appropriate	too high	n.a.
2.3 The amount of content in relation to the available time is:	○	○	○	○

3. Competency Development (I)

We would now like to assess to what extent the learning objectives of the Professional Identity Formation track have been achieved. The PIF sessions are intended to support development in the CanMEDS roles.

	very confide nt					very unsure	n.a.
3.1 Medical Expert<br>Medical Experts integrate all CanMEDS roles and apply medical knowledge, clinical skills, and professional judgment to provide high-quality, patient-centered care.	○	○	○	○	○	○	○

Please describe a situation during this year’s sessions or placements in which you acted as a Medical Expert.

	very confident					very unsure	n.a.
3..2 Scholar<br>Scholars demonstrate a lifelong commitment to learning, critically assess information, apply evidence-based knowledge, and contribute to the advancement of knowledge.	○	○	○	○	○	○	○
Please describe a situation during this year's sessions or placements in which you practiced the Scholar role.							

	very confident					very unsure	n.a.
3.3 Communicator<br>Communicators effectively exchange information with patients, families, and team members – fostering trust, understanding, and therapeutic relationships.	○	○	○	○	○	○	○
Please describe a situation during this year's sessions or placements in which you practiced the Communicator role.							

	very confident					very unsure	n.a.
3.4 Collaborator<br>Collaborators work effectively with other healthcare professionals to provide safe, high-quality, patient-centered care through teamwork and shared decision-making.	○	○	○	○	○	○	○
Please describe a situation during this year's sessions or placements in which you practiced the Collaborator role.							

PREVIEW
Do not fill in!

3. Competency Development (II)

	very confident					very unsure	n.a.
3.5 Health Advocate<br>Health Advocates contribute to the health and well-being of patients and communities by identifying needs and promoting supportive environments and policies.	○	○	○	○	○	○	○
Please describe a situation during this year's sessions or placements in which you practiced the Health Advocate role.							

	very confident					very unsure	n.a.
3.6 Leader<br>Leaders engage with others to contribute to the effectiveness of healthcare organizations, manage resources responsibly, and support quality improvement.	○	○	○	○	○	○	○
Please describe a situation during this year's sessions or placements in which you practiced the Leader role.							

	very confident					very unsure	n.a.
3.7 Professional<br>Professionals commit to the health and well-being of patients through ethical practice, professional accountability, and self-awareness.	○	○	○	○	○	○	○

Please describe a situation during this year's sessions or placements in which you practiced the Professional role.

4. Alignment with the Curriculum

To what extent do you agree with the following statements?
The PIF curriculum...

	applies	somewhat applies	rather does not apply	does not apply	n.a.
4.1 …is well integrated into the overall curriculum.	O	O	O	O	O
4.2 …helped me prepare for the observership(s) or block internships.	O	O	O	O	O
4.3 …helped me reflect on the observership(s) or block internships.	O	O	O	O	O

4.4 Which topics did you miss or would you like to see added in the current PIF year?

5. Design of the Sessions

	applies	somewhat applies	rather does not apply	does not apply	n.a.
5.1 The session was clearly structured.	O	O	O	O	O
5.2 The learning objectives were addressed.	O	O	O	O	O
5.3 There was opportunity to ask questions.	O	O	O	O	O
5.4 The atmosphere encouraged open discussion, including on personal issues.	O	O	O	O	O

Please note: Due to data protection regulations, feedback on individual tutors is not recorded in this evaluation. We kindly ask you to forward such feedback directly to the responsible module coordinators.

	applies	somewhat applies	rather does not apply	does not apply	n.a.
5.5 The tutor structured the session according to the PIF handbook.	O	O	O	O	O
5.6 The tutor appeared competent in the subject matter.	O	O	O	O	O

	too short	appropriate	too long	n.a.
5.7 The amount of time allocated for the session content was:	O	O	O	O

PREVIEW
Do not fill in!

6. Group Work

The work in the small group...

	applies	somewhat applies	rather does not apply	does not apply	n.a.
6.1 …provided a safe and confidential space for exchange.	O	O	O	O	O
6.2 …was characterized by respectful interaction.	O	O	O	O	O
6.3 …allowed sufficient room for reflection.	O	O	O	O	O

	too smal	appropriate	too big	n.a.
6.4 The group size was:	O	O	O	O

7. Evaluation of the Handbook

	applies	somewhat applies	rather does not apply	does not apply	n.a.
7.1 The handbook was helpful for my learning progress in medical school.	O	O	O	O	O
7.2 The task descriptions in the handbook were clearly formulated.	O	O	O	O	O
7.3 The handbook content fit well into this year of study.	O	O	O	O	O

7.4 Suggestions for improvement regarding the handbook:

8. Workshop Program (Year 5)

8.1 How many workshops from the Year 5 PIF series did you attend?	○ 0 ○ 1 ○ 2-3 ○ 4 ○ 5-8 ○ >8				
	applies	somewhat applies	rather does not apply	does not apply	n.a.
8.2 I am very satisfied with the thematic selection of the workshop series.	○	○	○	○	○
8.3 I am very satisfied with the scheduling of the workshops I attended.	○	○	○	○	○
8.4 I am very satisfied with the content structure of the workshops I attended.	○	○	○	○	○
8.5 The instructors...					
Please note: Due to data protection regulations, feedback on individual tutors is not recorded in this evaluation. We kindly ask you to forward such feedback directly to the responsible module coordinators.					
	applies	somewhat applies	rather does not apply	does not apply	n.a.
8.5.1 …appeared professionally competent.	○	○	○	○	○
8.5.2 …explained things clearly.	○	○	○	○	○
8.5.3 …were well prepared.	○	○	○	○	○
8.5.4 …actively involved students in the learning process.	○	○	○	○	○
8.5.5 …motivated me to engage more deeply with the topic.	○	○	○	○	○
8.6 The Year 5 PIF workshops appropriately complement the sessions from previous study years.	○	○	○	○	○
8.7 Overall, I find the concept of the Year 5 PIF workshop series to be good.	○	○	○	○	○
8.8 Additional suggestions or comments regarding the workshop series:<br>Please ensure that no conclusions can be drawn about individual persons.					

PREVIEW
Do not fill in!

9. Final Comments

We would like to know whether the topics addressed accompany you in everyday life, and what suggestions you have for improvement.						
	always	often	occasion-ally	rarely	never	n.a.
9.1 The topics addressed provide meaningful points of connection for my professional development, even beyond the PIF curriculum.	○	○	○	○	○	○

9.2 What I liked:
9.3 What I liked less and my suggestions for improvement:
9.4 What I still wish for:
